# Different experiences of two PRRT2-associated self-limited familial infantile epilepsy

**DOI:** 10.1007/s13760-020-01348-9

**Published:** 2020-04-03

**Authors:** Qianlei Zhao, Zhenwei Liu, Ying Hu, Shiyu Fang, Feixia Zheng, Xiucui Li, Feng Li, Zhongdong Lin

**Affiliations:** 1 Department of Pediatric Neurology, The Second Affiliated Hospital and Yuying Children’s Hospital of Wenzhou Medical University, Wenzhou, China; 2 Institute of Genomic Medicine, Wenzhou Medical University, Wenzhou, China; 3 Department of Pediatric, The First People’s Hospital of Aksu District, Xinjiang Uygur Autonomous Region, China

**Keywords:** Self-limited familial infantile epilepsy, Oxcarbazepine, PRRT2, Sodium channel blocker

## Abstract

To analyze the clinical characteristics and PRRT2 gene mutation of self-limited familial infantile epilepsy and evaluate the treatment responses of different antiepileptic drugs in self-limited familial infantile epilepsy. We reviewed the clinical feature and genetic mutation results and treatment responses of two sibling sisters. They were detected with the PRRT2 gene mutation through Sanger sequencing. Elder sister was treated with oxcarbazepine oral suspension, while younger sister was treated with levetiracetam oral solution. The two sibling sisters exhibited PRRT2 heterozygous mutation inherited from their mother in c.649dupC p.(Arg217fs). Oxcarbazepine oral suspension had an immediate effect on the elder sister who was treated with it. However, levetiracetam oral solution had no effect on younger sister even though the dose was increased, but she got seizure-free after turning to oxcarbazepine oral suspension. Oxcarbazepine, which plays the mechanism of the sodium channel blockers, has a more significant effect than levetiracetam, which has no mechanism of the sodium channel blockers in self-limited familial infantile epilepsy. The PRRT2 gene of infantile epileptic patients with a family history of infantile convulsions or paroxysmal kinesigenic dyskinesia(PKD) could be detected by sanger sequencing and a biomarker to select antiepileptic drugs which play the mechanism of the sodium channel blockers could be utilized.

## Background

Benign familial/non-familial partial seizures in infancy were first proposed by Watanabe et al., as benign partial epilepsy in infancy(BPEI) with complex partial seizures (CPS) or secondary generalised seizures (SGS) and Watanabe et al., also proposed a clinical entity of BPEI combining BPEI with CPS and BPEI with SGS [1–3]. Thereafter, patients reported by Vigevano et al. [4], namely “Benign Infantile Familial Convulsiosimilarns”. Then the reports of the disease increased gradually. In 2001, the International League Against Epilepsy (ILAE) proposed that benign familial infantile convulsions (BFIC) should be classified as an independent epilepsy syndrome and be classified as familial focal epilepsy, which is also known as benign familial infantile seizures (BFIS). In 2010, ILAE commission revised the terminology and concepts for organization of seizures and epilepsies and modified it to benign familial infantile epilepsy (BFIE) as an electroclinical syndrome [5]. At the same time, ILAE recommended the descriptive term ‘‘self-limited’’ instead of “benign” [5]. According to new terminology and definitions of 2017 ILAE classification of the epilepsies, the term “benign” should be replaced by “Self-limited” or “pharmacoresponsive”, because the term “benign” underestimate the impact of epilepsy’s comorbidities on an individual’s life [6]. At present, ILAE uses the term self-limited familial (and non-familial) infantile epilepsy [7], “Self-limited” referred to the likely spontaneous resolution of a syndrome [6]. In families with self-limited familial infantile epilepsy, some affected members may present paroxysmal choreoathetosis [8]. Therefore, infantile convulsions with paroxysmal choreoathetosis (ICCA) syndrome was proposed to describe benign infantile epilepsy (BIE) and dyskinesia appeared successively in one individual or different affected individuals in one family. Most of them were motor evoked, alternatively, paroxysmal kinesigenic dyskinesia (PKD).

Great progress has also been made in the study of pathogenic genes in self-limited familial infantile epilepsy. Chen et al. [9], using whole-exome sequencing followed by Sanger sequencing, identified that mutations within proline-rich transmembrane protein 2 (PRRT2) gene was related to PKD in Han Chinese families. Research also confirmed that more than 70% of children with self-limited familial infantile epilepsy and almost all patients with ICCA were related to PRRT2 gene mutations [10]. At present, PRRT2 gene mutations were considered to be the main cause of self-limited familial infantile epilepsy, PKD and ICCA. Therefore, the concept of PRRT2-associated paroxysmal diseases was proposed based on gene linkage analysis and common clinical characteristics [11]. Among different PRRT2 mutations, c.649dupC was so far the most common cause of self-limited familial infantile epilepsy [12]. Herein, we report different experiences of two PRRT2-associated self-limited familial infantile epilepsy.

## Methods

### PRRT2 gene detection method

The primers of PRRT2 gene (nm_145239.2) covering the target gene exons and their flanking regions for PCR were designed by Primer 3 (https://bioinfo.ut.ee/primer3-0.4.0/primer3/). Its primers consisted of 5′CTGAGACAGGAATGTGGCC3′ (sense) and 5′GTGGCTCAGAGGGTTAGGTC3′ (antisense) for Exon2, 5′CTTCACTCCTCCTTCCTCCC3′ (sense) and 5′CTGTAAACAAGGCCGCTCAG 3′ (antisense) for Exon3-4. The DNA sample of PRRT2 gene (exon and its flanking sequence) was amplified by Taq DNA polymerase kit. After amplification, the electrophoresis bands were clear and specific, and Sanger sequencing was performed by the Life 3500 Dx sequencing instrument. Sequencing data is analyzed with Mutation Surveyor Software, and judge the pathogenicity of genetic mutation according to literature and several databases (including 1000 Genome, ExAC, ESP, HGMD, ClinVar, etc.).

### Ethics

Informed consent was obtained from the parents legal representative and patients were enrolled after their eligibility assessment. This study was approved by the Independent Ethics Committees and Institutional Review Board of the Second Affiliated Hospital and Yuying Children’s Hospital, Wenzhou Medical University (No.LCKY2018-11), and was conducted according to the ethical principle of the Declaration of Helsinki.

## Results

### Clinical manifestations

Two sibling sisters were diagnosed with self-limited familial infantile epilepsy according to Report of the ILAE Classification Core Group. Their mother and mother’s younger half-brother had a history of seizure in infancy but had no formal treatment. Both of them were in remission within 2 years old and outcome was favorable. Elder sister was diagnosed at the age of 10 months in our hospital in July 2010, younger sister was diagnosed at 3 months old in another hospital in September 2018 and transferred to our hospital at 6 months old in December 2018. Both of them presented focal seizure and/or GTCS. Attacks presented with motor arrest, slow deviation of the head and eyes to one side, generalized hypertonia, cyanosis, synchronous or asynchronous limb tonic–clonic jerks, first started unilateral, then became bilateral and seizures may occur in several clusters per day, for a period varying from 1 to 4 days, most attacks occur during the awake period. Both background of the ictal video-EEG were normal. Cranial MRI were normal. Psychomotor and neurologic development were normal before and after the episodes.

### Genetic findings

**Fig. 1:**
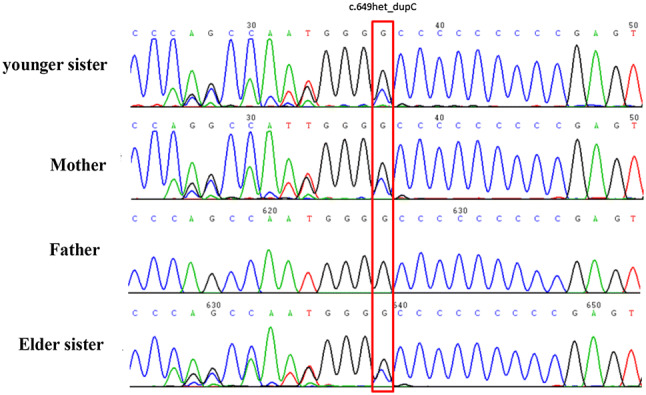
PRRT2 heterozygous mutation in c.649dupC p.(Arg217fs)

Elder sister did not take PRRT2 gene test in time after diagnosis. Because at that time, the relationship between self-limited family infantile epilepsy and PRRT2 was not clear. Younger sister took PRRT2 gene detection about 3 months after diagnosis when she was transferred from another hospital to our hospital. At the same time, we tested the elder sister's PRRT2 genes, even though she was 9 years old and got seizure-free several years ago. The result showed that both of them had PRRT2 heterozygous mutation in c.649dupC p.(Arg217fs) which inherited from their mother(Fig. 1). The patients with a positive family history were characterized by an autosomal dominant trait.

### Treatment responses

The elder sister was treated with oxcarbazepine (OXC) oral suspension with an initial dosage of 1.5 ml twice daily (60 mg/ml, 15 mg/kg days) after diagnosis in our hospital at the age of 10 months (weight 12 kg). Consequently, OXC had an effect and she got seizure-free immediately. We tested the serum concentration of active metabolite of OXC after 2 weeks of treatment in the morning before taking the drug and the value was 10.1 μg/ml. The OXC dose is constant during the treatment. The outcome of elder sister was favorable with no developmental delay and cognitive impairment and she stopped taking OXC at 3 years old without recurrence during the following more than 7 years. However, younger sister was initially treated with levetiracetam (LEV) oral solution with an initial dosage of 0.6 ml twice daily (100 mg/ml, 20 mg/kg days) in another hospital at 3 months old (weight 6 kg), but it had no effect. The dosage of LEV was increased gradually every month by 20 mg/kg daily until the maximum recommended dose of 60 mg/kg daily in more than 1 month. Unfortunately, seizure still could not be controlled. But she got seizure-free immediately after turning to OXC oral suspension at the initial dosage of 0.75 ml twice daily (60 mg/ml, 10.59 mg/kg days) after transferring to our hospital at 6 months old (the weight was 8.5 kg at that time). The serum concentration of active metabolite of OXC was tested after 2 weeks treatment of OXC, the value was 6.5 μg/ml and we did not increase the dosage. We reduced the dosage in follow-up after 1 year (18 months old, weight was 11 kg), from 0.75 ml twice daily (8.18 mg/kg days) to 0.50 ml twice daily (5.45 mg/kg days) and the seizure did not recur and the psychomotor development of young sister was also normal. We are planning to reduce the dose to 0.25 ml twice daily (2.73 mg/kg days) and stop OXC at 2 years old. It’s worth mentioning that it is not necessary to increase the dose of OXC with the weight gaining of infants younger than 1 year of age because low dosage OXC could still continue to control the seizures.

## Discussion

The clinical characteristics of self-limited familial infantile epilepsy are clusters of brief focal seizures onset of 3–12 months after birth and can progress to secondary generalized seizures. The genetic pattern of self-limited familial infantile epilepsy is autosomal dominant inheritance with imperfectness of penetrance and has genetic heterogeneity. Among genes associated with self-limited familial infantile epilepsy, PRRT2 gene is the most important pathogenic gene of self-limited familial infantile epilepsy [12–14] and the mutational hot spots are c.649dupc and c.649delc, among which c.649dupc is the commonest mutation leading to self-limited familial infantile epilepsy [12], which is consistent with the cases reported in this paper.

Despite the lack of large-scale research evidence, existing reports indicate that common PRRT2 mutations are more likely to get remission through carbamazepine treatment and the effective dosages might be much lower than it used to treat epilepsy [11]. PKD patients generally respond rapidly to anticonvulsants, including carbamazepine, phenytoin or other anticonvulsive, such as valproate, oxcarbazepine, lamotrigine, levetiracetam and topiramate. Yang et al. [15] compared the efficacy and tolerability of OXC and CBZ in the treatment of PKD patients aged 13–33 years, the results showed that both of them can markedly reduce the attack frequency and the degree of reduction are similarly. Pan et al. [16] suggested low doses of OXC in the range 5–20 mg/kg days as draughts in the morning can be an effective treatment option for paediatric PKD patients, for 19 patients stopping dyskinesia attacks in total 20 patients. Zhang et al. [17] reported a PRRT2 heterozygous mutation in a three generations Chinese family with ICCA and PKD and found oxcarbazepine/phenytoin treatment was dramatically effective for them.

Self-limited familial infantile epilepsy is a self-limiting seizure disorder with favorable outcome. In most cases, a single anticonvulsant can completely control the attacks and a combined anticonvulsants treatment is rarely necessary. Zhou et al. [18] reported 15 children aged 4 months–15 years old with PRRT2-associated paroxysmal diseases, among them, 2 cases went into remission through the first choice of valproate treatment. However, in 11 cases who treated with levetiracetam as the first choice, 6 cases achieved remission, but 5 cases were not well controlled and had to add or replace with valproate, topiramate or oxcarbazepine for control. Ebrahimi-Fakhari et al. [11] analyzed the pharmacotherapy of self-limited familial infantile epilepsy, the results showed that the top three anticonvulsants were phenobarbital, carbamazepine and valproate. We report two PRRT2-associated self-limited familial infantile epilepsy of sibling sisters in a family, both of them were in rapid remission through oxcarbazepine treatment, but not levetiracetam. It suggests that PRRT2-associated self-limited familial infantile epilepsy has a better response to oxcarbazepine than levetiracetam, which may be related to the blocking mechanism of sodium channel.

## Conclusion

Antiepileptic drugs (Oxcarbazepine Oral Suspension) which play the mechanism of the sodium channel blockers have a more significant curative effect for PRRT2-associated self-limited familial infantile epilepsy. For patients with a family history of self-limited familial infantile epilepsy, PKD or ICCA, the PRRT2 gene could be detected by sanger sequencing and used as a biomarker for the selection of anticonvulsants.
